# Computed Tomography Pulmonary Perfusion for Prediction of Short-Term Clinical Outcome in Acute Pulmonary Embolism

**DOI:** 10.1055/s-0041-1723782

**Published:** 2021-02-10

**Authors:** Lisette F. van Dam, Lucia J. M. Kroft, Menno V. Huisman, Maarten K. Ninaber, Frederikus A. Klok

**Affiliations:** 1 Department of Thrombosis and Hemostasis, Leiden University Medical Center, Leiden, The Netherlands; 2 Department of Radiology, Leiden University Medical Center, Leiden, The Netherlands; 3 Department of Pulmonology, Leiden University Medical Center, Leiden, The Netherlands

**Keywords:** pulmonary embolism, diagnosis, diagnostic imaging, perfusion, prognosis

## Abstract

**Background** Computed tomography pulmonary angiography (CTPA) is the imaging modality of choice for the diagnosis of acute pulmonary embolism (PE). With computed tomography pulmonary perfusion (CTPP) additional information on lung perfusion can be assessed, but its value in PE risk stratification is unknown. We aimed to evaluate the correlation between CTPP-assessed perfusion defect score (PDS) and clinical presentation and its predictive value for adverse short-term outcome of acute PE.

**Patients and Methods** This was an exploratory, observational study in 100 hemodynamically stable patients with CTPA-confirmed acute PE in whom CTPP was performed as part of routine clinical practice. We calculated the difference between the mean PDS in patients with versus without chest pain, dyspnea, and hemoptysis and 7-day adverse outcome. Multivariable logistic regression analysis and likelihood-ratio test were used to assess the added predictive value of PDS to CTPA parameters of right ventricle dysfunction and total thrombus load, for intensive care unit admission, reperfusion therapy and PE-related death.

**Results** We found no correlation between PDS and clinical symptoms. PDS was correlated to reperfusion therapy ( *n* = 4 with 16% higher PDS, 95% confidence interval [CI]: 3.5–28%) and PE-related mortality ( *n* = 2 with 22% higher PDS, 95% CI: 4.9–38). Moreover, PDS had an added predictive value to CTPA assessment for PE-related mortality (from Chi-square 14 to 19, *p* = 0.02).

**Conclusion** CTPP-assessed PDS was not correlated to clinical presentation of acute PE. However, PDS was correlated to reperfusion therapy and PE-related mortality and had an added predictive value to CTPA-reading for PE-related mortality; this added value needs to be demonstrated in larger studies.

## Introduction

Computed tomography pulmonary angiography (CTPA) is the current imaging modality of choice for the diagnosis of pulmonary embolism (PE). 1 In recent years, technical advances have been made in the diagnostic management of PE including the introduction of computed tomography pulmonary perfusion (CTPP) imaging. With CTPP additional information of hemodynamic and functional impact of the PE as expressed by measures of pulmonary perfusion can be assessed. 2

Available studies using CTPP have mostly focused on its diagnostic performance for acute PE. The addition of CTPP to CTPA has been reported to improve the specificity for a PE diagnosis 3 and to improve the detection rate of small, subsegmental emboli. 4 5 Also, perfusion defects on CTPP were found to be correlated to PE thrombus load and signs of right ventricular dysfunction on CTPA. 6 7 8 9 10 Therefore, perfusion defects on CTPP may be relevant for prognostication of PE patients as well, although this is less well studied. For instance, the quantification of perfusion defects may predict PE-related death, hemodynamic collapse, or need for oxygen therapy. This information is relevant for initial risk stratification and treatment or to consider home treatment in patients with good prognosis. 11

In this study, we aimed to evaluate the correlation between perfusion defects on CTPP and clinical symptoms at presentation and its predictive value for adverse short-term outcome of acute PE.

## Patients and Methods

### Study Design and Population

This was a prospective observational study in a convenience sample of 100 consecutive hemodynamically stable adult patients (≥18 years) with CTPA-confirmed acute symptomatic PE, diagnosed between July 2017 and October 2019 in the Leiden University Medical Center in whom CTPP was performed as part of routine clinical practice. Patients were excluded in case of nonassessable CTPP scan due to imaging artifacts. The diagnostic management of patients with suspected acute PE started with assessment of the clinical pre-test probability in combination with D-dimer testing, following the YEARS algorithm. 12 13 In patients with CTPA-confirmed acute PE, anticoagulant treatment was started or modified in patients already on anticoagulant treatment according to international standards. The Hestia rule, consisting of 11 clinical criteria, was used to identify low-risk PE patients for outpatient treatment. 11 14 15 This study was approved by the institutional review board of the Leiden University Medical Center, and informed consent requirement was waived due to its observational nature.

### Primary and Secondary Aim

The primary aim was to investigate the correlation between quantification of CTPP-measured perfusion defects with clinical symptoms at presentation and its predictive value for adverse short-term 7-day outcome. The secondary aim was to investigate the added value of CTPP reading to right ventricle to left ventricle diameter ratio (RV/LV ratio), pulmonary artery trunk diameter and total thrombus load on CTPA for prediction of intensive care unit (ICU) admission, reperfusion therapy, and PE-related mortality. Furthermore, the correlation between PDS on CTPP and total thrombus load on CTPA was evaluated.

### Outcomes

For the primary outcome, clinical symptoms at presentation and adverse short-term outcome were evaluated. Clinical symptoms included (non)pleural chest pain, dyspnea, and hemoptysis. Adverse short-term outcome included hospital or ICU admission, need for supplemental oxygen therapy or intravenous pain medication >24 hours, reperfusion therapy, vasopressor or inotropic therapy, and PE-related death within 7-day follow-up. All symptoms and outcomes were assessed from digital patient files.

For the secondary outcome, we assessed prognostic imaging signs on CTPA including RV/LV ratio, pulmonary artery trunk diameter, and total thrombus load. The predictive capacity of these CTPA clinical imaging signs and of PDS for the outcome of ICU admission, reperfusion therapy, and PE-related mortality was evaluated.

### Image Acquisition and Analysis

**Fig. 1:**
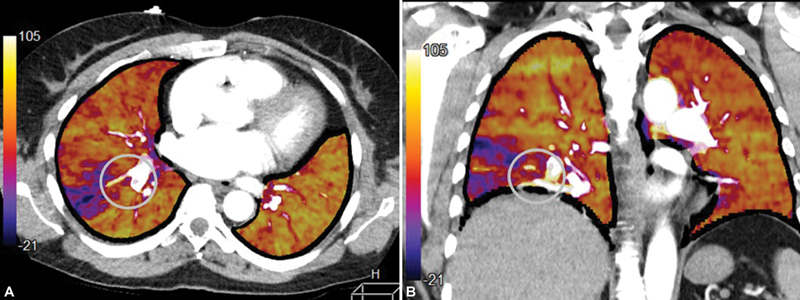
( **A** ) Fused parametric perfusion map with computed tomography pulmonary angiography, axial and ( **B** ) coronal image in a patient with an acute thrombus in the right lower lobe pulmonary artery (encircled) with subsegmental reduced lung perfusion in the laterodorsal segment of the right lower lobe.

Since June 2017, CTPP is part of the standard CT angiography protocol in adult patients with suspected PE at our hospital. CT examinations were performed on a 320-multislice detector row CT scan (Canon). CTPP images were acquired by using subtraction technique, in which the precontrast image is subtracted from the contrast-enhanced image. The subtraction image is then color coded and fused with the CTPA images; normal perfusion: yellow to orange, moderately decreased perfusion: red to pink, and severely decreased or absent perfusion: purple to dark blue/black ( Fig. 1 ).

For this analysis, CTPP and CTPA image reading was performed independently by two different readers, who were unaware of presenting symptoms and occurrence of adverse events. CTPP assessment was performed by a researcher (L.F.V.D) trained by an expert thoracic radiologist (L.J.M.K.). Perfusion defects were quantified per segment by using the score proposed by Chae et al and expressed as mean PDS in percentage. 10 To assess the interobserver agreement for PDS reading, CTPP images of 25 consecutive patients were independently evaluated by a second reviewer (L.J.M.K.). RV/LV ratio, pulmonary artery trunk diameter, and total thrombus load on CTPA were evaluated by one expert thoracic radiologist (L.J.M.K.) with over 20 years of experience in pulmonary CTPA reading. The maximum diameters of both the right and left ventricle were measured in the standard axial view with measurement of the maximal distance between the ventricular endocardium and the interventricular septum. The pulmonary artery trunk was measured at its largest transverse diameter. The total thrombus load was assessed by using the CT obstruction index according Qanadli et al. 16

### Definitions

Acute PE was defined as at least one filling defect in the pulmonary artery tree on CTPA. 17 18 19 Pleural chest pain was defined as sharp chest pain that worsens during breathing. Nonpleural chest pain was defined as pressure on or squeezing sensation in the chest. PE-related death was defined as objectively confirmed clinically severe PE before death in the absence of an alternative diagnosis. 20

### Statistical Analysis

Baseline characteristics are described as mean with standard deviation (SD) or median with interquartile range. To evaluate the correlation between PDS to clinical symptoms and adverse outcomes, the difference between the mean PDS with corresponding 95% confidence interval (CI) in patients with versus without chest pain, dyspnea, and hemoptysis and adverse short-term outcome was calculated. To evaluate the agreement in PDS scoring between the two reviewers, the mean difference between PDS assessed by reviewer 1 and 2 was calculated.

The added predictive value of PDS to CTPA assessment for ICU admission, reperfusion therapy, and PE-related death was assessed by comparing two prediction models. In the first prediction model, CTPA parameters including RV/LV ratio, pulmonary artery trunk diameter, and total thrombus load were included. In the second prediction model, PDS assessment was added to these CTPA parameters. Multivariable logistic regression analysis and the likelihood-ratio test were performed to assess the predictive value of the two models for ICU admission, reperfusion therapy, and PE-related death and whether PDS assessment significantly improved the predictive value of the model. Additionally, to quantify the performance of the prediction models, we determined the discrimination and calibration. Discrimination refers to the ability to discriminate between those with and those without the outcome and calibration to the agreement between observed outcomes and predictions. Discrimination was expressed with the concordance (c) statistic, by calculating the area under the receiver operating characteristic curve (AUC) with a 95% CI, with discrimination considered perfect if AUC of 1, good if AUC >0.8, moderate if AUC 0.6 to 0.8, poor if AUC <0.6, and no better than chance if AUC = 0.5. Calibration was assessed by using the Brier score, which ranges from 0 to 0.25, with a score of zero signifies a perfect prediction model and a score of 0.25 a noninformative model. 21

The correlation between PDS and total thrombus load was evaluated by using the Pearson's correlation test. A two-sided *p* -value of *p* <0.05 was considered as statistical significant. All statistical analyses were performed in SPSS version 25 (IBM, Armonk, New York, United States).

## Results

### Study Population

A total of 100 patients with CTPA proven acute PE were eligible for analysis. The baseline characteristics of the 100 included patients are shown in Table 1 . Four patients were transferred to another hospital within 48 hours because of logistical reasons. Results for adverse short-term outcome were thus available for 96 patients. The mean PDS of all included patients was 27% (SD = 13%) and mean Qanadli score was 30% (SD = 23%). Forty-nine patients (49%) had a RV/LV ratio >1 ( Table 1 ). The agreement in PDS scoring between the two reviewers was good with a mean difference in PDS of 4.2% (SD = 6.9%).

**Table 1:** Baseline characteristics of 100 patients with acute pulmonary embolism

Mean age (±SD), y	62 (16)
Male, *n* (%)	53 (53)
Median duration of complaints (IQR), d	2.0 (1–7)
Recurrent VTE, *n* (%)	17 (17)
Active malignancy *, n* (%)	27 (27)
Immobility for >3 d or recent long travel >6 h in the past 4 weeks *, n* (%)	24 (24)
Trauma/surgery during the past 4 wk *, n* (%)	22 (22)
Active inflammation/infection	3 (3)
Hormone (replacement) therapy *, n* (%)	7 (7)
Known genetic thrombophilia *, n* (%)	0 (0)
Outpatient	80 (80)
Mean PDS score (±SD), %	27 (13)
Mean Qanadli score (±SD), %	30 (23)
RV/LV ratio > 1 *, n* (%)	49 (49)

Abbreviations: IQR, interquartile range; PDS, perfusion defect score; RV/LV ratio, right ventricle to left ventricle diameter ratio; SD, standard deviation; VTE, venous thromboembolism.

### Primary Outcome

The prevalence of symptoms at presentation and adverse short-term outcome with associated mean PDS are presented in Table 2 . Of the 100 patients, dyspnea was present in 84 (84%), pleural chest pain in 55 (55%), nonpleural chest pain in 25 (25%) and hemoptysis in 6 patients (6.0%). A total of 60 patients (60%) were admitted to the hospital, of whom 7 patients (7.3%) were admitted to the ICU. Twenty-five patients (26%) were treated with oxygen >24 hours, 6 patients (6.3%) received intravenous pain medication >24 hours, 4 patients (4.2%) received reperfusion therapy, and 3 patients (3.1%) needed vasopressor/inotropic therapy. We did not find a relevant correlation between PDS and clinical presentation ( Table 2 ). The PDS was associated with reperfusion therapy (16% higher PDS, 95% CI: 3.5–28%) and PE-related mortality (22% higher PDS, 95% CI: 4.9–39%; Table 2 ). The PDS was not associated with the need for oxygen therapy, pain medication, or vasopressor/inotropic therapy nor with ICU admission.

**Table 2:** Perfusion defect score in 100 acute pulmonary embolism patients and correlation to presenting symptoms and short-term adverse outcome

	Prevalence (%)	Mean (SD) PDS in % in patients with:	Mean (SD) PDS in % in patients without:	Difference (95% CI)
Symptoms at presentation ( *n* = 100) and 7-day outcome ( *n* = 96)				
Pleural chest pain a	55 (55)	28 (14)	27 (12)	1.5 (−3.7 to 6.7)
Nonpleural chest pain a	25 (25)	32 (12)	26 (13)	5.5 (−0.41 to 11)
Dyspnea	84 (84)	28 (13)	25 (13)	3.4 (−3.7 to 11)
Hemoptysis	6 (6.0)	27 (14)	27 (13)	−0.74 (−12 to 10)
Hospital admission	60 (60)	28 (14)	27 (11)	0.92 (−4.4 to 6.2)
ICU admission	7 (7.3)	32 (19)	26 (12)	5.7 (−3.9 to 15)
Oxygen therapy >24 h	25 (26)	28 (15)	26 (12)	1.4 (−4.3 to 7.2)
IV pain medication >24 h	6 (6.3)	23 (12)	27 (12)	−4.6 (−15 to 5.7)
Reperfusion therapy	4 (4.2)	42 (14)	26 (12)	16 (3.5–28) b
Need for vasopressor therapy	3 (3.1)	34 (32)	27 (12)	7.6 (−6.8 to 22)
PE-related death	2 (2.1)	49 (19)	27 (12)	22 (4.9–39) b

Abbreviations: CI, confidence interval; CTPP, computed tomography pulmonary perfusion; ICU, intensive care unit; PDS, perfusion defect score; PE, pulmonary embolism; SD, standard deviation.

a Patients could have either pleural chest pain or nonpleural chest pain, or both at the same time.

b Symptoms/outcome correlated to PDS on CTPP.

### Secondary Outcome

The results from logistic regression and likelihood-ratio test are shown in Table 3 . The first model including CTPA parameters alone was correlated to ICU admission (Chi-square [χ ^2^ ] = 17.1, degrees of freedom [df] = 3, *p* = 0.001). Model 2 was also correlated to ICU admission (χ ^2^ = 17.9 df = 4, *p* = 0.001), but the predictive capacity hardly improved when PDS was added to CTPA parameters (χ ^2^ = 0.799, df = 1, *p* = 0.371). Model 1 including CTPA parameters and model 2 with addition of PDS assessment were both able to predict reperfusion therapy (χ ^2^ = 20.9, df = 3, *p* < 0.001 and χ ^2^ = 24.1, df = 4, *p* < 0.001, respectively). However, the predictive capacity of the model did not improve when PDS was added to CPTA parameters (χ ^2^ = 3.22, df = 1, *p* = 0.073). Model 1 including CTPA parameters alone was able to predict PE-related mortality (χ ^2^ = 13.8, df = 3, *p* = 0.003). Model 2 was also able to predict PE-related mortality (χ ^2^ = 19.3, df = 4, *p* = 0.001) and the addition of PDS scoring also increased the predictive capacity of the model (χ ^2^ = 5.44, df = 1, *p* = 0.020). The odds ratios including 95% CI of each CTPA parameter and PDS within prediction model 2 for each adverse outcome are provided in Table 3 .

**Table 3:** Predictive value, area under the receiver operating characteristic curve and Brier score of model 1 (CTPA parameters including RV/LV ratio, pulmonary artery trunk diameter, and total thrombus obstruction score) and model 2 (CTPA parameters and perfusion defect score) and odds ratios for each parameter within model 2 for ICU admission, reperfusion therapy, and PE-related mortality

	Chi-square	df	Sign.	AUC (95% CI)	Brier score	Odds ratio (95% CI)
ICU admission						
Model 1	17.1	3	0.001	0.852 (0.647–1.00)	0.042	
Model 2 • RV/LV ratio • Pulmonary artery trunk diameter • Total thrombus obstruction score • Perfusion defect score	17.9	4	0.001	0.876 (0.725–1.00)	0.043	6.27 (0.880–44.6) 1.03 (0.832–1.28) 1.07 (0.999–1.15) 0.955 (0.859–1.06)
Difference between model 1 and 2	0.799	1	0.371			
Reperfusion therapy						
Model 1	20.9	3	<0.001	0.976 (0.938–1.00)	0.021	
Model 2 • RV/LV ratio • Pulmonary artery trunk diameter • Total thrombus obstruction score • Perfusion defect score	24.1	4	<0.001	0.984 (0.951–1.00)	0.013	65.4 (0.243–1.76 ^E^ + 4) 1.51 (0.866–2.63) 1.21 (0.975–1.49) 1.19 (0.947–1.49)
Difference between model 1 and 2	3.22	1	0.073			
PE-related mortality						
Model 1	13.8	3	0.003	0.989 (0.965–1.00)	0.010	
Model 2 • RV/LV ratio • Pulmonary artery trunk diameter • Total thrombus obstruction score • Perfusion defect score	19.3	4	0.001	1.00 (1.00–1.00)	<0.001	Not applicable due to low number of events
Difference between model 1 and 2	5.44	1	0.020			

Abbreviations: AUC, area under the receiver operating characteristic curve; CI, confidence interval; Df, degrees of freedom; ICU, intensive care unit; RV/LV ratio, right ventricle to left ventricle diameter ratio.

The AUC for CTPA parameters to predict ICU admission was 0.852 (95% CI: 0.647–1.00), 0.976 (95% CI: 0.938–1.00) for reperfusion therapy and 0.989 (95% CI: 0.965–1.00) for PE-related mortality. When PDS was added to the prediction model, these AUCs were 0.876 (95% CI: 0.725–1.00), 0.984 (95% CI: 0.951–1.00), and 1.00 (95% CI: 1.00–1.00), respectively ( Table 3 ). The prediction model including CTPA parameters had a Brier score of 0.042 for ICU admission, 0.021 for reperfusion therapy, and 0.010 for PE-related mortality. Prediction model 2 had a Brier score of 0.043 for predicting ICU admission, 0.013 for reperfusion therapy, and <0.001 for PE-related mortality ( Table 3 ).

With the use of the Pearson correlation test, a positive correlation between the total PDS and CTPA-assessed total thrombus load was found ( *r* = 0.523, *p* < 0.001).

## Discussion

We showed that perfusion defects on CTPP are correlated to reperfusion therapy and PE-related mortality, and that the addition of PDS assessment to CTPA assessment of RV/LV ratio, pulmonary artery trunk diameter, and total thrombus load improved the predictive value of the model to predict PE-related mortality, but not ICU admission nor reperfusion therapy. Moreover, perfusion defects on CTPP did not correlate to clinical symptoms at presentation.

Risk stratification of patients with acute PE is crucial for deciding on the optimal treatment, including hospitalization, close hemodynamic monitoring, and reperfusion therapy. 1 22 23 Previous studies found that right ventricle enlargement (RV/LV ratio > 1.0) is associated with an increased risk for PE-related mortality. 24 25 26 Current European guidelines therefore recommend the assessment of right ventricular dimensions or function as part of initial risk stratification. 22 As previous publications have shown that CTPP-assessed PDS is correlated to RV/LV ratio and total thrombus load, 6 7 8 9 10 perfusion imaging may play a role in this risk stratification. Although our results showed an improvement in the predictive capacity for PE-related mortality when PDS was added to CTPA-reading, the improvement in AUC was only marginal. Furthermore, we could not confirm an added value of PDS over CTPA assessment to predict ICU admission nor reperfusion therapy. A possible explanation may be the low incidence of these adverse outcomes (range between two and seven patients).

We also evaluated whether perfusion defects on CTPP were correlated to clinical symptoms at presentation. This is relevant, as pain and dyspnea for which treatment with intravenous pain medication, and oxygen therapy may be needed are also relevant for the decision for hospitalization or home treatment. 27 28 However, an association between PDS and presenting symptoms could not be established. Of note, as the generation of dyspnea and chest pain involve multiple underlying (complex and not fully understood) mechanisms, a discrepancy between chest pain and dyspnea and extent of perfusion defects in acute PE is possible. 29 PDS was also not correlated to hospital admission. However, the decision to admit a patient to the hospital is often based on multiple variables, some not included in this analysis, including pregnancy, active bleeding and the presence of a social reason for treatment in hospital.

In current literature, the addition of CTPP to CTPA was found to improve the specificity in the PE detection from 94% (95% CI: 89–97%) to 100% (95% CI: 100–100%) and the detection of occlusive (sub)segmental pulmonary emboli. 30 CTPP was also evaluated for PE prognostication and was shown to be correlated to adverse clinical outcome including ICU admission, all-cause and PE-related mortality, 7 31 but had no added value to RV/LV ratio to predict mortality. 32 33 Hence, based on our results and available literature, the application of CTPP seems to be mostly relevant for the diagnostic management of acute PE rather than for prognostication.

Limitations of the study are its observational design and the use of a convenience cohort without a specific sample size calculation. This latter may have resulted that the study was underpowered to detect a correlation between PDS and clinical symptoms and some adverse outcomes. On the other hand, the predictive value of perfusion defects for reperfusion therapy and PE-related mortality may be overestimated due to the low incidence of these adverse events and should therefore be interpreted with caution. Furthermore, the presence of clinical symptoms was self-reported and not assessed in a standardized manner, what may have introduced relevant bias. Bias may also be present in the perfusion defect quantification as perfusion defects may not only be the result of a PE but also of other pathology such as pneumonia. The strengths of this study are its prospective design and the inclusion of all comers, which supports the external validity of our findings. Also, CTPA and CTPP assessment was performed by independent readers who were unaware of the clinical presentation and course.

In conclusion, PDS was not associated with clinical presentation of acute PE. However, our data showed that CTPP-assessed PDS was correlated to reperfusion therapy and PE-related mortality and improved the predictive value of CTPA reading for PE-related mortality, but not for ICU admission or reperfusion therapy. Due to the limited number of adverse events and the design or our study, our observations should be considered hypothesis generating. Future larger studies including an upfront determined sample size calculation are needed to determine the clinical relevance of PDS quantification on top of CTPA assessment of right ventricle dysfunction in risk stratification of acute PE.
